# Intelligent rehabilitation in an aging population: empowering human-machine interaction for hand function rehabilitation through 3D deep learning and point cloud

**DOI:** 10.3389/fncom.2025.1543643

**Published:** 2025-05-02

**Authors:** Zhizhong Xing, Zhijun Meng, Gengfeng Zheng, Guolan Ma, Lin Yang, Xiaojun Guo, Li Tan, Yuanqiu Jiang, Huidong Wu

**Affiliations:** ^1^School of Rehabilitation, Kunming Medical University, Kunming, China; ^2^Medical Imaging Key Laboratory of Sichuan Province, North Sichuan Medical College, Nanchong, China; ^3^Fujian Key Laboratory of Special Intelligent Equipment Safety Measurement and Control, Fujian Special Equipment Inspection and Research Institute, Fuzhou, China; ^4^School of Pharmaceutical Science and Yunnan Key Laboratory of Pharmacology for Natural Products, Kunming Medical University, Kunming, China; ^5^School of Mechanical Engineering, Xi'an Jiaotong University, Xi'an, China; ^6^Xi'an Aerospace Automation Co., Ltd, Xi'an, China; ^7^School of Mechanical and Automotive Engineering, South China University of Technology, Guangzhou, China

**Keywords:** 3D perception, neural network, human-machine interaction, deep learning, non-contact rehabilitation

## Abstract

Human-machine interaction and computational neuroscience have brought unprecedented application prospects to the field of medical rehabilitation, especially for the elderly population, where the decline and recovery of hand function have become a significant concern. Responding to the special needs under the context of normalized epidemic prevention and control and the aging trend of the population, this research proposes a method based on a 3D deep learning model to process laser sensor point cloud data, aiming to achieve non-contact gesture surface feature analysis for application in the field of intelligent rehabilitation of human-machine interaction hand functions. By integrating key technologies such as the collection of hand surface point clouds, local feature extraction, and abstraction and enhancement of dimensional information, this research has constructed an accurate gesture surface feature analysis system. In terms of experimental results, this research validated the superior performance of the proposed model in recognizing hand surface point clouds, with an average accuracy of 88.72%. The research findings are of significant importance for promoting the development of non-contact intelligent rehabilitation technology for hand functions and enhancing the safe and comfortable interaction methods for the elderly and rehabilitation patients.

## 1 Introduction

In the context of the ongoing normalization of global epidemic prevention and control measures and the accelerating aging of the population, the dual challenge of safeguarding public health and enhancing the well-being of the elderly and rehabilitation patients has emerged as a pressing issue in contemporary society (Huang et al., 2023; Baraković et al., 2024; Lopes et al., 2023; Harada et al., 2021; Li L. et al., 2023). Especially in the field of intelligent rehabilitation of hand functions in human-machine interaction, traditional rehabilitation methods are limited by contact based operations and infection risks, making it difficult to meet current needs (An et al., 2022; Gao et al., 2023; Jiang et al., 2021; Zhang et al., 2023; Samhan et al., 2020; Tang et al., 2025). Therefore, developing a non-contact, efficient, and accurate gesture recognition system is of great significance for promoting the development of intelligent rehabilitation technology for human-machine interaction hand function.

There are many limitations in the field of hand function rehabilitation (Gu et al., 2022; Edger-Lacoursière et al., 2023; Zestas and Tselikas, 2023; Ergen et al., 2024; Bates and Sunderam, 2023). Traditional hand function rehabilitation methods are mostly contact based, such as therapists manually assisting patients with hand movement training. This approach relies on the manpower input of professional therapists, and in the face of the growing population of elderly rehabilitation patients, treatment resources are often difficult to meet the demand. In addition, existing rehabilitation assessment methods largely rely on the subjective judgment of therapists and lack objective and accurate data support. When evaluating the accuracy and flexibility of hand function, there may be differences in the evaluation criteria of different therapists, resulting in lower reliability of the evaluation results. The non-contact gesture recognition method proposed in this research avoids direct contact between patients and treatment equipment or therapists. At the same time, it can achieve intelligent gesture analysis and recognition, reduce reliance on professional therapists’ manpower, improve the efficiency of rehabilitation treatment, and better meet the needs of elderly rehabilitation patients. By analyzing the hand surface point cloud, our proposed method can extract rich hand features and provide accurate data support for rehabilitation assessment.

Different sensors can be used to obtain hand surface information. LEAP device plays an important role in fields such as human-machine interaction and hand motion capture (Vaitkevičius et al., 2019; Ameur et al., 2020; Najafinejad and Korayem, 2023; Li et al., 2019; Galván-Ruiz et al., 2020). The LEAP device mainly utilizes optical imaging principles to obtain 3D coordinate information of the hand, with performance advantages of high frame rate and high accuracy, and can track subtle hand movements in real time. In the application of virtual reality, game development, and scientific research, LEAP equipment played a crucial role. LEAP device performs well in indoor environments with stable lighting and close range operations. What’ more, MediaPipe, as a powerful and widely used open-source framework, has achieved remarkable results in the field of pose detection (Suwabe et al., 2024; Cao et al., 2024; Samaan et al., 2022; Mariappan et al., 2024). With excellent performance, it can accurately recognize hand movements and postures, and has outstanding application performance in many scenarios. MediaPipe combines various advanced algorithms, greatly promoting the development of computer vision and human-machine interaction fields, and providing important support for related research and applications. In addition, laser sensors, as a high-precision, non-contact measurement tool, have been widely used in the fields of material surface morphology analysis, defect detection, and so on (Zheng et al., 2023; Ye et al., 2023; Rufei et al., 2022; Sadaoui et al., 2022; Theodose et al., 2021). The point cloud data collected by laser sensors can obtain 3D structural information of material surfaces, providing important basis for material performance analysis and interface engineering. Meanwhile, laser sensor point clouds, as a non-contact gesture acquisition technology, can obtain 3D information of the hand with high precision and high spatiotemporal resolution, providing a rich data source for gesture analysis. However, in the field of human-machine interaction and intelligent rehabilitation of hand functions, the application of laser sensor point cloud data is still relatively limited. Therefore, this research aims to apply laser sensor point cloud data to gesture recognition, process point cloud data through a 3D deep learning model, and achieve non-contact gesture surface feature analysis.

There are various methods in the field of point cloud processing, among which the point cloud processing method based on multi view method is widely used (Zhang et al., 2018; Tong et al., 2020). This method projects three-dimensional point cloud data onto multiple two-dimensional views, and then uses traditional two-dimensional image processing techniques for analysis and processing. Its advantage is that it can use mature two-dimensional image processing algorithms to reduce processing difficulty. However, this method inevitably loses some three-dimensional spatial information during the projection process, resulting in damage to the integrity of point cloud data and limiting subsequent analysis. The point cloud processing based on voxel method divides the three-dimensional space into regular voxel grids (Li J. et al., 2021; Li Y. et al., 2021; Aljumaily et al., 2023), which fills the point cloud data into corresponding voxels, and then uses a three-dimensional convolutional neural network for processing. This method can effectively preserve the spatial structure information of point cloud data. However, high-resolution voxels can lead to an exponential increase in computational complexity, while low resolution voxels cannot accurately express the detailed features of point clouds. PointNet and PointNet++ break through the limitations of point cloud processing methods (Qi et al., 2017a; Qi et al., 2017b). As deep learning models capable of directly processing point cloud data, they break the traditional convolutional neural network’s dependence on structured data. PointNet and PointNet++ can effectively extract global features of point clouds by introducing symmetric functions, demonstrating good performance in tasks such as point cloud classification and object detection.

The continuous emergence of efficient solutions for upper limb motor performance evaluation, hand rehabilitation, human-computer interaction, and other excellent achievements (Guo et al., 2023; Yozevitch et al., 2023; Zhu et al., 2025; Lu et al., 2024). We have achieved a lot of research results based on graph neural network in the early stage (Xing et al., 2023; Xing et al., 2025; Xing et al., 2022), but non-contact gesture surface feature analysis for intelligent rehabilitation of hand function in human-machine interaction is still blank. Moreover, due to the complexity and diversity of gesture surface features, how to effectively extract and analyze gesture surface features from point cloud data has become a challenging problem.

In summary, this research combines laser sensor point cloud data with 3D deep learning model to construct an efficient and accurate gesture recognition system in the context of normalized epidemic prevention and control and aging population trends. Through this system, we can achieve precise analysis of the surface features of gestures, providing new solutions for intelligent rehabilitation of hand functions in human-machine interaction. This research achievement can provide a non-contact gesture analysis tool for rehabilitation medical institutions and elderly care institutions, reduce the risk of infection transmission, improve the efficiency and accuracy of rehabilitation treatment, and promote the cross integration of interface science and deep learning technology, opening up new paths for research in related fields. In addition, this method can also be applied in fields such as augmented reality and gesture control interfaces, providing users with a more natural and intuitive way of human-machine interaction.

## 2 Materials and methods

**Figure 1:**
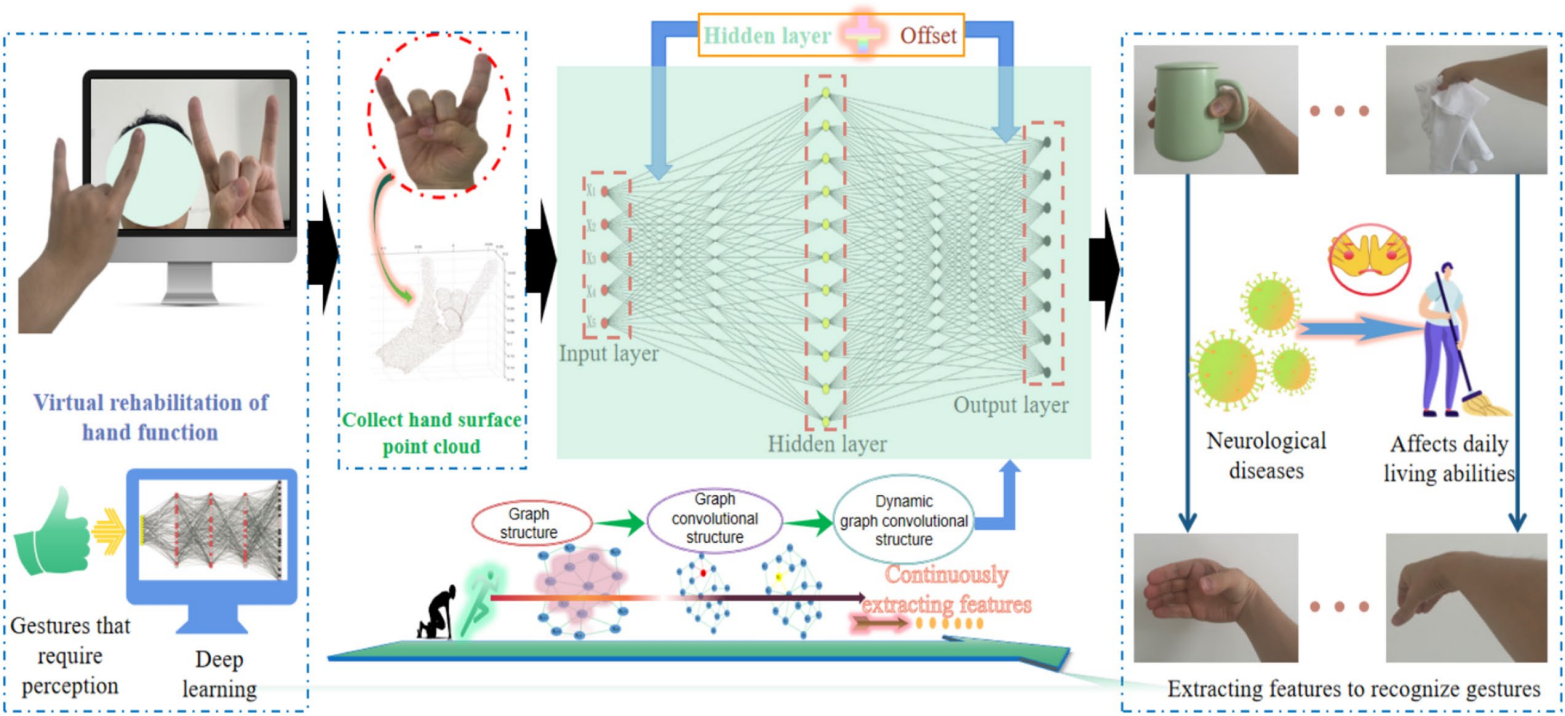
Building a research framework based on 3D deep learning.

Gesture recognition is a crucial step in achieving natural human-machine interaction in hand function virtual rehabilitation systems (Li W. et al., 2023; Liu et al., 2022; Wang et al., 2023; Zhu et al., 2021). Due to the high degree of freedom of human hand joints, achieving precise and robust breakthroughs in gesture recognition technology is currently a challenging task. A point cloud refers to a collection of points on the surface characteristics of a target object, which have 3D coordinates and simple expression methods (Tan et al., 2022; Xiao et al., 2023; Akhtar et al., 2022), making it easy to express spatial features of different gestures in a digital form. More importantly, point cloud data is generally obtained through non-contact measurement devices such as laser sensors. This research builds a gesture surface feature analysis network (GSFAN) based on DGCNN to revolutionize the research approach of gesture recognition in 3D space (Wang et al., 2019), as shown in Figure 1.

### 2.1 Hand 3D data

**Figure 2:**
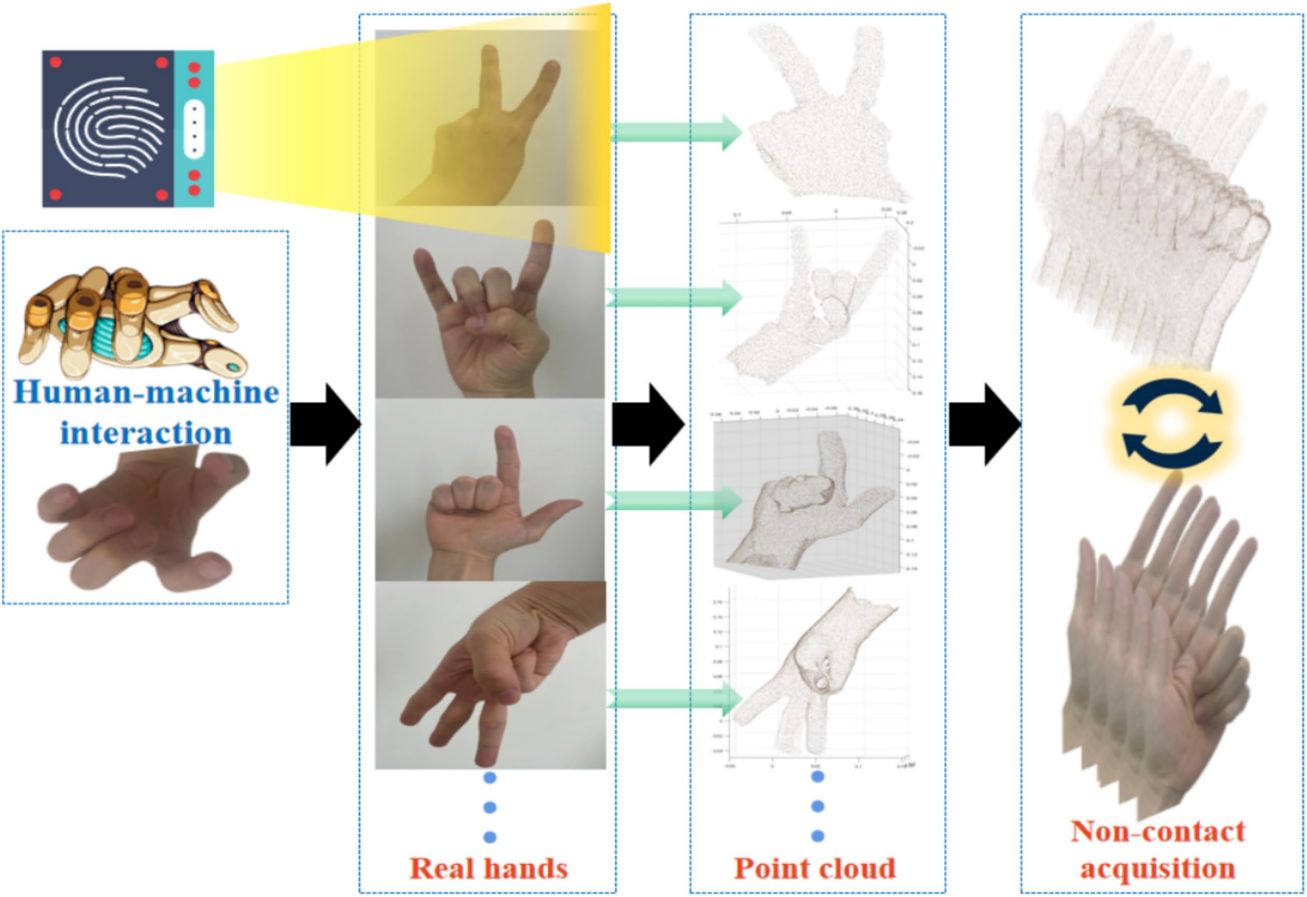
Collecting 3D data of hand surface for non-contact human-machine interaction.

The collection of hand surface point clouds is the primary step in this research. By collecting point cloud representations of hand surface data, it provides a foundation for subsequent analysis of hand surface features and functional rehabilitation. In order to obtain 3D information of the hands, we used light laser detection and ranging as a data acquisition device to obtain point cloud data of the hands. In the process of collecting point clouds on the surface of the hand, choose an environment with sufficient light and no obstructions, while ensuring that the background of the hand collection area forms a clear contrast with the hand, in order to better extract the features of the hand. In this research, we collected point clouds on the surface of the hands of different individuals to ensure data diversity. When collecting hand surface point clouds, different individuals displayed different postures in order according to regulations, as shown in Figure 2.

The total number of participants is 4. The distance between the sensor and the hand is not fixed, which facilitates us to quickly and conveniently collect hand surface point cloud. Although variable distances can increase noise, we can use CloudCompare to remove it. In the actual data collection process, the technology used by laser radar is Direct Time of Flight (DToF). The main reasons for choosing laser radar are as follows: Firstly, laser radar has the advantage of non-contact, which avoids the risk of cross infection that may be caused by contact devices. Secondly, laser radar has high-precision characteristics and can capture subtle structures of the hand. Finally, the anti-interference ability of laser radar enables stable acquisition of depth information even under complex lighting conditions. Therefore, laser radar plays a crucial role in the entire process of collecting hand surface point cloud. It can accurately capture hand surface features and generate high-quality point cloud data, which provides a solid data foundation for our research. This research sets each finger (thumb, index finger, middle finger, ring finger, little finger) as an independent variable, and each finger is in two states of extension and bending. A total of 32 gestures were calculated through combination.

After the data collection was completed, we adopted a series of steps to process the raw point cloud data to remove irrelevant information. Firstly, we will import the collected point cloud data into the professional point cloud processing software CloudCompare, which has powerful visualization and processing capabilities, allowing us to intuitively view the quality and distribution of point cloud data. Secondly, after importing the data, we checked if there is any interference from other objects around the hand surface point cloud. If there are non-hand surface point cloud, we used CloudCompare’s segmentation tool to process them. Specifically, we combined the software visualization interface to accurately select the hand surface point cloud data to be preprocessed in the point cloud data list, ensuring accurate operation. Next, we used segmentation tools to draw a closed shape that fully includes the non-hand surface point cloud that needs to be deleted. We further utilized the segmentation tool sub option to separate non hand surface point cloud from the original point cloud data, and used the deletion function of the point cloud data list to completely delete the separated point clouds. Finally, after completing the above processing operations, we stored the processed hand surface point cloud data.

### 2.2 Hand surface point cloud edge convolution for human-machine interaction

**Figure 3:**
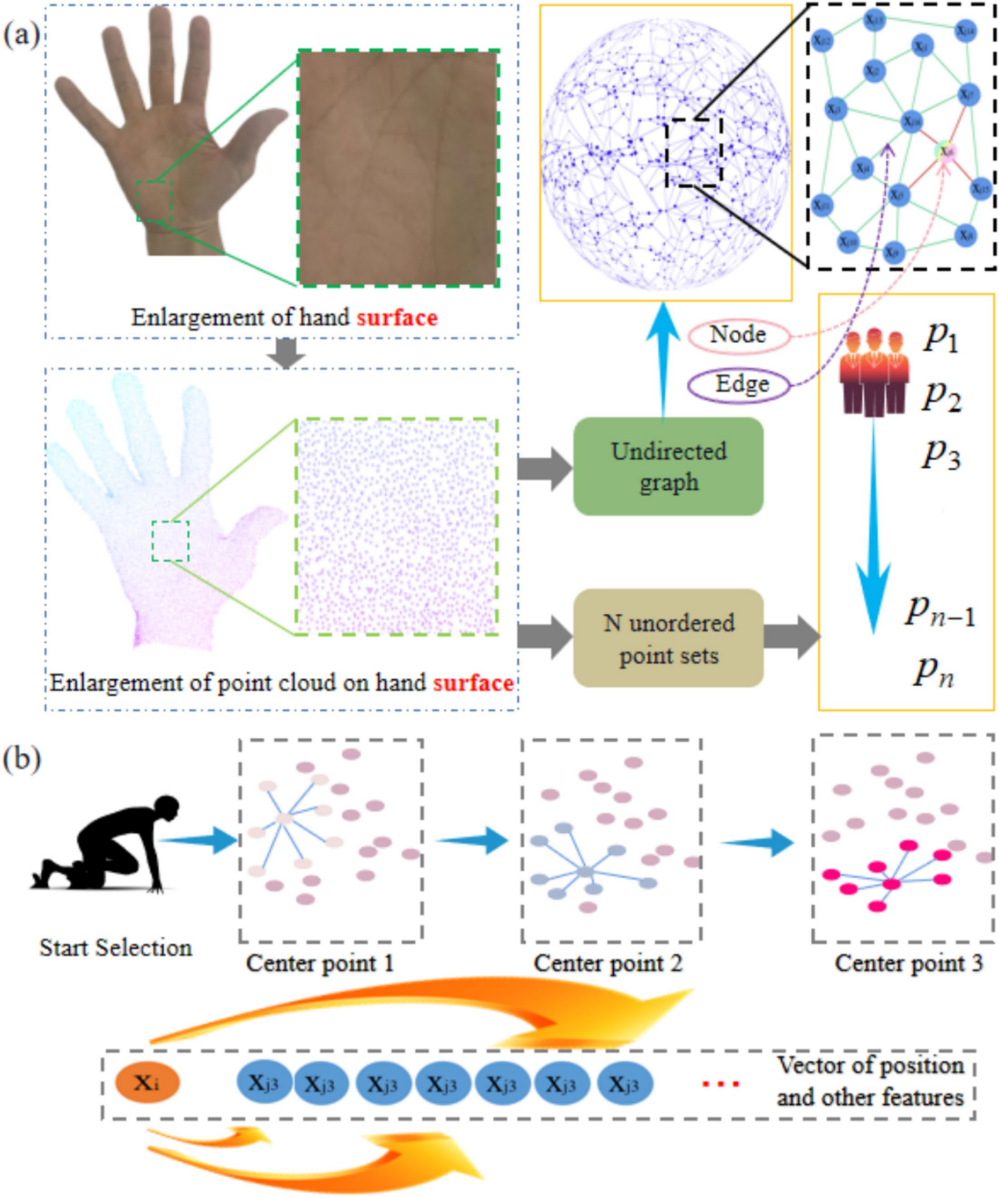
**(a)** Graph structure representation of real hand surface point clouds. **(b)** Each point in the hand surface point clouds is traversed as the center point.

In GSFAN, edge convolution is a key technique that can effectively capture local structures and geometric features in hand surface point cloud data. The point cloud on the surface of the hand can be viewed as an unordered set of N points, thus it can be represented as an undirected graph, where each point is a node in the graph, and the edges between nodes represent their proximity (Figure 3a). In edge convolution, it is necessary to construct a graph structure to capture local proximity information of the hand surface point cloud. The commonly used methods for constructing graphs include different algorithms such as radius neighbors, which can determine the connection relationship based on the distance between each center point and its nearest neighbor point. Once the graph structure is constructed, edge convolution operations can be performed. The basic idea of edge convolution is to update the node representation by aggregating the features of neighboring nodes of each node. In the point cloud of the hand surface, each node can be represented as a vector containing position and other features. The edge convolution operation obtains a new representation of each node by aggregating and updating the features of its neighboring nodes (Figure 3b). GSFAN extracts higher-level feature representations by stacking multiple layers of edge convolution, gradually learning richer hand surface point cloud features. However, whether the more edge convolution layers, the better the effect, will be analyzed in detail and depth in this research.

The node set V contains all the points in the hand surface point cloud. Each point $v_{i}$ corresponds to a coordinate point in three-dimensional space, and the set of node V can be expressed as Equation (1).


(1) $$V=\left\{v_{1},v_{2},\ldots v_{N}\right\}$$

Where N is the number of points in the point cloud on the surface of the hand. Each node $v_{i}$ can be represented by a feature vector $x_{i}$ , which contains the coordinates and other relevant information of the point, and can be expressed as Equation (2).


(2) $$x_{i}=\left\{x_{i}^{\mathrm{coord}},x_{i}^{\mathrm{other}}\right\}$$

Where $x_{i}^{\mathrm{coord}}$ is the three-dimensional coordinates of the point (for example, $x_{i}=\left[x,y,z\right]$ ), and $x_{i}^{\mathrm{other}}$ is other features such as color and normal.

The edge set E defines the connection relationship between nodes in the graph, and for hand surface point clouds, threshold distance is used to define edges. For any two nodes $v_{i}$ and $v_{j}$ , if the spatial distance between them is less than a certain threshold $d_{\mathrm{thresh}}$ , it can be expressed as Equation (3).


(3) $$E=\left\{e_{ij}|\mathrm{dist}\left(vi,vj\right)<d_{\mathrm{thresh}}\right\}$$

Where $\mathrm{dist}\left(vi,vj\right)$ is a function for calculating the distance between nodes $v_{i}$ and $v_{j}$ . In order to more conveniently represent the graph structure, this research uses the adjacency matrix A , which can be expressed as Equation (4).


(4) $$A=\left[\begin{array}{cccc} A_{11} & A_{12} & \cdots & A_{1N}\\ A_{21} & A_{22} & \cdots & A_{2N}\\ \vdots & \vdots & \ddots & \vdots \\ A_{N1} & A_{N2} & \cdots & A_{NN} \end{array}\right]$$

If there is an edge between nodes $v_{i}$ and $v_{j}$ , $A_{ij}=1$ , otherwise $A_{ij}=0$ . In this research, we assume that there is a hand surface point cloud dataset of P , where each point $p_{i}$ has a 3D coordinate $\left(x_{i},y_{i},z_{i}\right)$ . For point $p_{i}$ , its local density can be expressed as Equation (5).


(5) $$p_{i}=\frac{1}{\left|N_{k}\left(p_{i}\right)\right|}\underset{p_{j}\in N_{k}\left(p_{i}\right)}{\sum }d\left(p_{i},p_{j}\right)$$

Where $N_{k}\left(p_{i}\right)$ is the set of K-nearest neighbors of point $p_{i}$ , and $d\left(p_{i},p_{j}\right)$ is the Euclidean distance between points $p_{i}$ and $p_{j}$ . The neighborhood $N\left(p_{i}\right)$ of point $p_{i}$ is defined as the set of all points up to $p_{i}$ that do not exceed $r_{i}$ , which can be expressed as Equation (6).


(6) $$N\left(p_{i}\right)=\left\{p_{j}\in P|d\left(p_{i},p_{j}\right)\leq r_{i}\right\}$$

Where $r_{i}$ is the neighborhood radius of each point. We use the weight matrix W and the nonlinear activation function σ to obtain the aggregated feature $h_{i}$ , which can be expressed as Equation (7).


(7) $$h_{i}=\sigma \left(W\cdot \mathrm{aggregate}\left(\left\{x_{j}|j\in N\left(p_{i}\right)\cup \left\{i\right\}\right\}\right)\right)$$

In order to enable each node to consider its own characteristics, we add self-loops and normalize them during the aggregation process, which can be expressed as Equation (8).


(8) $$h_{i}=\sigma \left(W\cdot \frac{1}{\left|N\left(p_{i}\right)+1\right|}\underset{j\in N\left(p_{i}\right)\cup \left\{i\right\}}{\sum }x_{j}\right)$$

Where $\left|N\left(p_{i}\right)\right|$ represents the number of nodes in the neighborhood $N\left(p_{i}\right)$ .

Traditional point cloud processing methods, such as simple voxelization or direct feature extraction, which often struggles to accurately capture local geometric structure information in point cloud data. Taking voxelization as an example, it divides the three-dimensional space into regular voxel grids and discretizes point cloud data into these grids. Although this approach reduces the complexity of the data to a certain extent, it also loses a large amount of local detail information. Edge convolution can fully consider the relationship between each point and its neighboring points when processing hand surface point cloud. Through edge convolution operation, the model can learn the local geometric structure in point cloud data based on the relative position between the center point and neighboring points.

In addition, traditional point cloud processing methods typically use fixed feature extraction methods, which may lack adaptability for different types of point cloud data. Edge convolution is different, it is a dynamic feature extraction method. In edge convolution, the features of each point are obtained by aggregating the features of its neighboring points. This feature extraction method enables edge convolution to learn more representative features based on the local structure and feature distribution of point cloud data. When processing hand surface point cloud, different gestures may have different local feature distributions. Edge convolution can adjust the feature extraction method based on these differences, thereby improving the expression ability of features and better distinguishing different gestures.

### 2.3 Enhancement of hand surface point cloud dimension abstraction in human-machine interaction

In hand surface point cloud recognition methods, improving the dimensional information of the point cloud helps to capture richer hand surface features and enhance the recognition ability of the model. Multilayer perceptron, as a feedforward neural network, can effectively handle nonlinear problems and abstract the intrinsic features of data (Yu et al., 2023; Lyu et al., 2022; Yang and Fang, 2024; Khan et al., 2022). Therefore, after edge convolution, we introduce a multilayer perceptron to further process hand surface point cloud data. After each fully connected layer, a multilayer perceptron follows an activation function to introduce nonlinear transformations. By stacking multiple such layers, multilayer perceptron can learn complex mappings from low to high dimensions and extract advanced features of hand surface point clouds.

**Figure 4:**
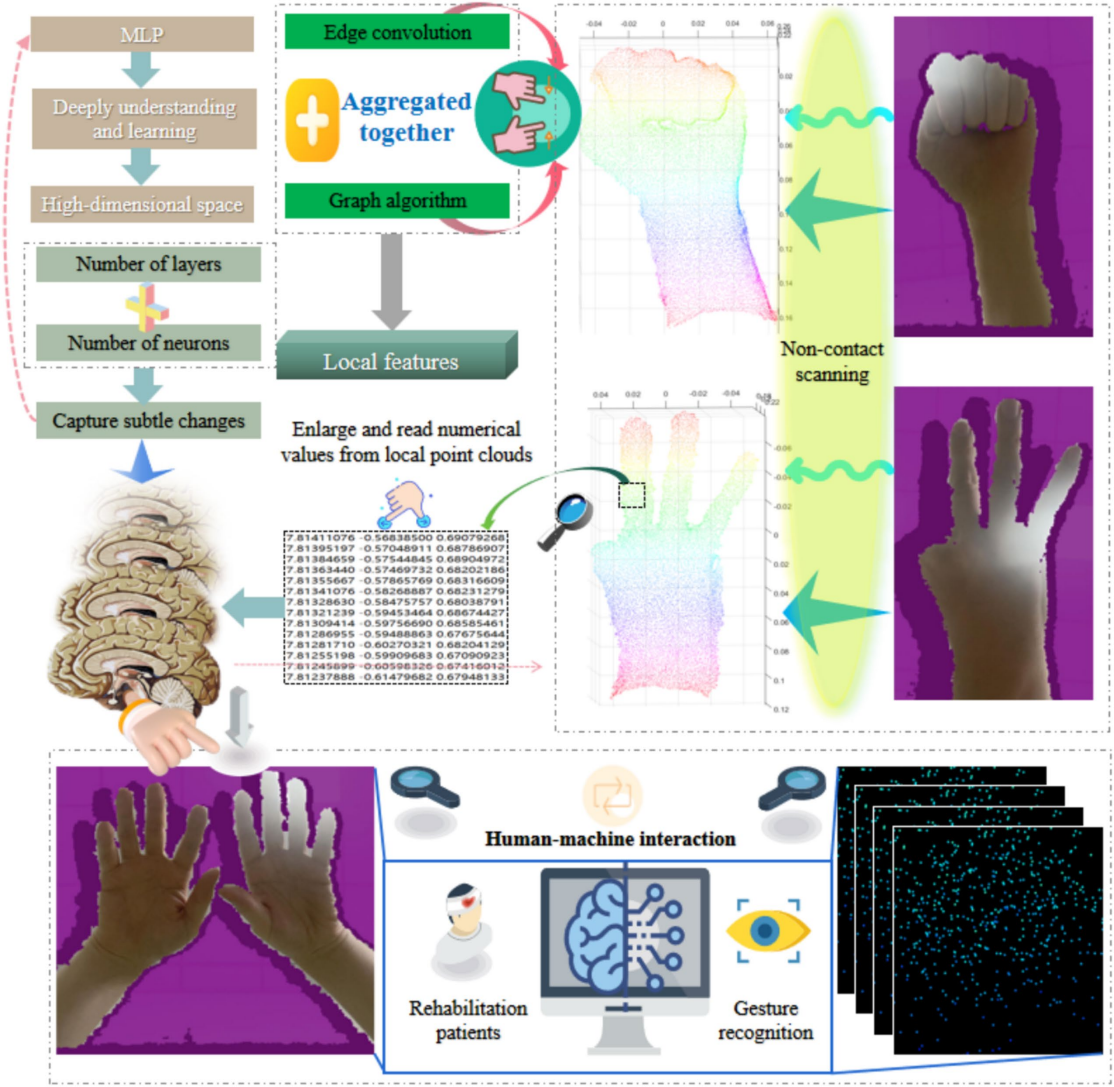
Abstract enhancement method for hand surface point cloud dimension in non-contact human-machine interaction.

Before inputting gesture point cloud data into a multilayer perceptron, we first perform preliminary processing on the gesture point cloud using edge convolution and graph algorithms to obtain local features for each point. Then, we use these local features as inputs to the multilayer perceptron, and gradually abstract the global features of the gesture point cloud through layer by layer transmission and transformation. An important feature of multilayer perceptron is its ability to enhance the dimensionality of data. By increasing the number of fully connected layers and neurons, we can map the input low dimensional features to a high dimensional space, thereby extracting more complex feature representations. The above process is shown in Figure 4. In hand surface point cloud recognition, this dimension enhancement helps to capture subtle changes and diversity in gestures, improving the recognition accuracy of GSFAN.

After edge convolution and KNN graph algorithm processing, the local feature representation of each hand surface point cloud can be obtained. Set $F=\left\{f_{1},f_{2},\ldots ,f_{N}\right\}$ to represent the local feature set of the hand surface point cloud, where $f_{i}\in R^{D_{0}}$ , N is the number of points, and $D_{0}$ is the initial feature dimension.

For the i output feature of the l layer in a multilayer perceptron, it can be expressed as Equation (9).


(9) $$h_{i}^{\left(l\right)}=\sigma \left(\overset{D_{i-1}}{\underset{j=1}{\sum }}\omega_{ij}^{\left(l\right)}h_{j}^{\left(l-1\right)}+b_{i}^{\left(l\right)}\right)$$

Where $h_{i}^{\left(l\right)}$ is the feature vector of the i row of the output matrix $H^{\left(l\right)}$ in the l layer, $\omega_{ij}^{\left(l\right)}$ is the element of the i row and j column of the weight matrix $W^{\left(l\right)}$ , $h_{j}^{\left(l-1\right)}$ is the vector composed of the features of the j column of the output matrix $H^{\left(l-1\right)}$ in the l−1 layer. $b_{i}^{\left(l\right)}$ is the *i*-th element of the bias vector $b^{\left(l\right)}$ . In order to obtain the output matrix $H^{\left(l\right)}$ of the entire l layer, we need to perform the above calculation on each $i\in \left\{1,2,\ldots ,D_{l}\right\}$ and stack the results in matrix form, which can be expressed as Equation (10).


(10) $$H^{\left(l\right)}=\sigma \left(W^{\left(l\right)}H^{\left(l\right)}+1_{N}⊗b^{\left(l\right)}\right)$$

Where $1_{N}$ is a full 1-column vector of N×1 , ⊗ represents Kronecker product or outer product, used to extend the bias vector $b^{\left(l\right)}$ to the same shape as $H^{\left(l-1\right)}$ for element wise addition. The complete matrix $H^{\left(0\right)}$ can be expressed as Equation (11).


(11) $$H^{\left(0\right)}=\left[\begin{array}{cccc} \sum_{j=1}^{N}\omega_{1j}\cdot M\left(j\cdot 1\right) & \sum_{j=1}^{N}\omega_{1j}\cdot M\left(j\cdot 2\right) & \ldots & \sum_{j=1}^{N}\omega_{1j}\cdot M\left(j\cdot D_{0}\right)\\ \sum_{j=1}^{N}\omega_{2j}\cdot M\left(j\cdot 1\right) & \sum_{j=1}^{N}\omega_{2j}\cdot M\left(j\cdot 2\right) & \ldots & \sum_{j=1}^{N}\omega_{2j}\cdot M\left(j\cdot D_{0}\right)\\ \vdots & \vdots & \ddots & \vdots \\ \sum_{j=1}^{N}\omega_{Nj}\cdot M\left(j\cdot 1\right) & \sum_{j=1}^{N}\omega_{Nj}\cdot M\left(j\cdot 2\right) & \ldots & \sum_{j=1}^{N}\omega_{Nj}\cdot M\left(j\cdot D_{0}\right) \end{array}\right]$$

In the formula, M is the mapping function. We introduce a combination of additional nonlinear functions, matrix multiplication, and element wise multiplication, which can be expressed as Equation (12).


(12) $$\tilde{z}=V\cdot \left[\sigma \left(z\right)⊙\tanh\left(W_{2}\cdot z+\beta \right)+\gamma \right]$$

Where V and $W_{2}$ are weight matrices, β and γ are additional learnable parameters, $\sigma \left(z\right)$ is the result of applying non-linear functions to z , $\tanh\left(W_{2}\cdot z+\beta \right)$ is the result of non-linear transformation, ⊙ represents element wise multiplication.

## 3 Results and discussions

### 3.1 Experimental details

We divided the collected hand surface point cloud dataset into training and testing sets for GSFAN training and evaluation. In order to ensure a certain degree of randomness and representativeness in the training and evaluation data during the experimental process, and to maintain a balance between different gesture categories in the dataset, in order to avoid GSFAN bias caused by class imbalance.

In this research, we used accuracy to evaluate the model. Choosing accuracy for evaluation is because it has significant advantages. In the gesture recognition scenario of hand function rehabilitation for the elderly population that we focus on, the accuracy can quickly demonstrate the overall reliability of the model for gesture recognition in practical applications, providing a strong basis for judging whether the model can meet basic needs. In addition, we also use tools such as confusion matrices to visualize and analyze the classification results of the model, in order to further research the performance of the model.

We not only conducted multiple repeated experiments, but also, due to different patients undergoing hand function rehabilitation at home, GSFAN needs to face diverse hand surface point clouds. Therefore, we collected the hand surface point clouds of different experimenters to test GSFAN and fully demonstrate its generalization. The configuration for conducting relevant experiments in this section is as follows: the processor is the 13th Gen Intel Core i9-13900 K, with a reference frequency of 3.00GHz and a maximum acceleration frequency of 5.80GHz. The graphics card is an NVIDIA GeForce RTX 4080 independent graphics card, with a core frequency of 2,205 MHz, a graphics memory frequency of 1,400 MHz, and a boost frequency of 2,505 MHz. The memory configuration consists of two 32GB DDR5 memory modules. The operating system is Windows 10 64 bit. In addition, different cutting-edge deep neural networks were constructed using Python 3.7.9 and TensorFlow 2.3.0 deep learning frameworks. The key hyperparameter settings are shown in Table 1.

**Table 1:** Hyperparameter settings.

Hyperparameters	Learning rate	Batch size	Momentum	Dropout rate	Loss function
Select	0.001	32	0.9	0.5	Cross-entropy

Although the ReLU function solves the problem of vanishing gradients, when x < 0, the gradient becomes 0, making the neuron invalid and not updated in the subsequent training process. Leaky ReLU does not use the method of all zeros in non-positive parts, but assigns a non-zero slope in non-positive parts (Wang and Liu, 2024; Gezawa et al., 2023). Therefore, Leaky ReLU is selected as the activation function.

### 3.2 Multi perspective analysis of edge convolution results

The number of edge convolution layers plays a crucial role in capturing local geometric features and learning discriminative representations of input point cloud data. In order to research the effect of different edge convolution layers, we conducted a series of experiments, and the number of edge convolution layers gradually increased from one layer. In all experiments, the remaining parts of the network and parameter settings were kept consistent to ensure the fairness of the experiment.

**Figure 5:**
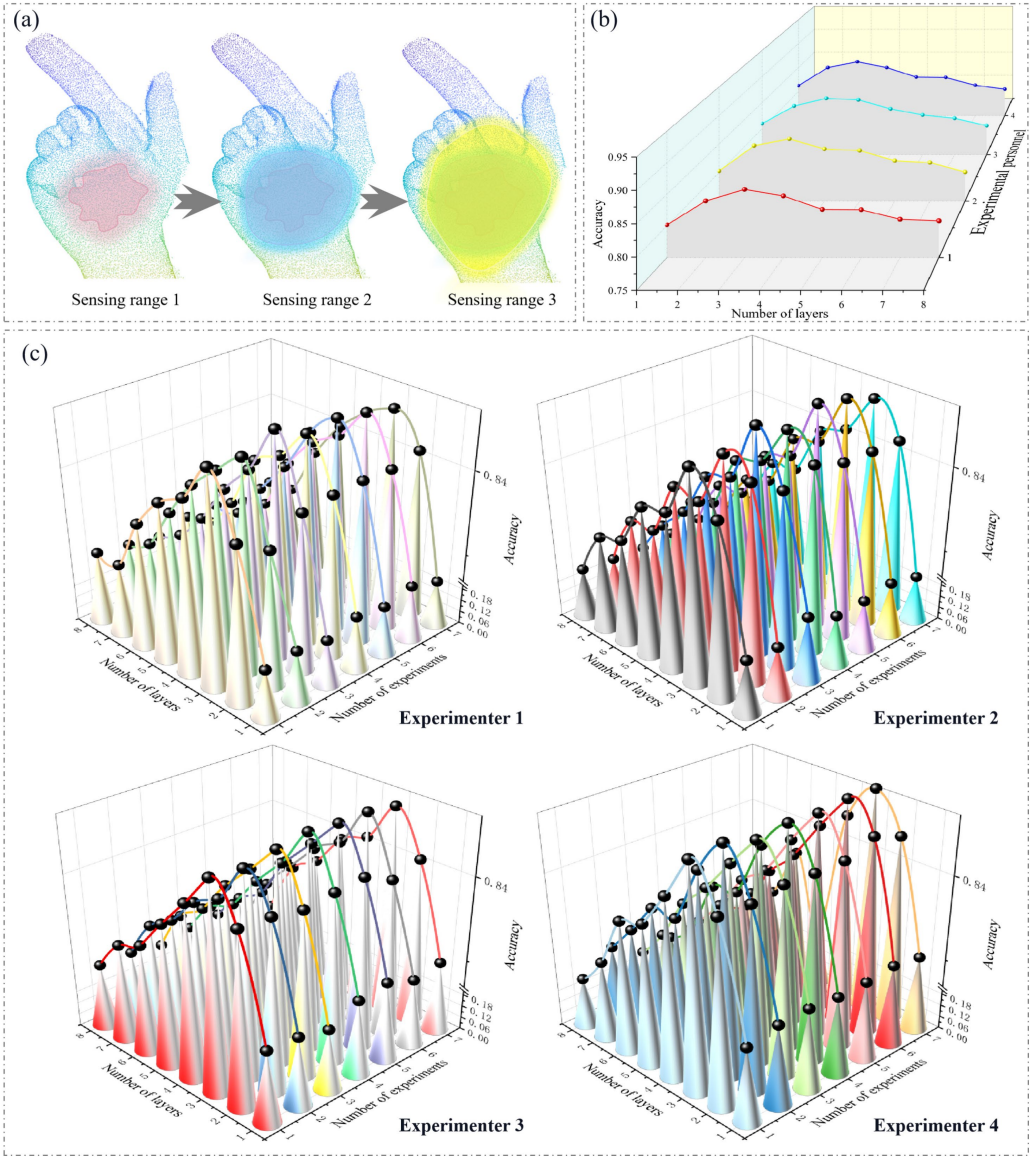
**(a)** Different receptive field ranges of hand surface point clouds. **(b)** Average values of experimenters in multiple experiments under different numbers of edge convolution layers. **(c)** Results of each experiment conducted by each experimenter under different numbers of edge convolution layers.

The more edge convolution layers attached within GSFAN, the larger the receptive field range of the hand surface point cloud, as shown in Figure 5a. We analyzed the results obtained by four experimenters under different numbers of edge convolution layers. In order to ensure the comprehensiveness and objectivity of the experiment, the results of each experiment were the average of multiple repeated experiments, as shown in Figure 5b. The experimental results of each experimenter under different numbers of edge convolution layers are shown in Figure 5c.

The performance of hand surface point cloud recognition methods is significantly affected by different numbers of edge convolution layers. We can see from the experimental results that as the number of edge convolution layers increases, the performance of hand surface point cloud recognition gradually improves. Starting from the number of edge convolution layers being 1, the accuracy gradually increases. Appropriately increasing the number of edge convolution layers can effectively improve the model’s ability to extract hand surface point cloud features, thereby enhancing the accuracy of gesture recognition. The edge convolution operation is crucial in the model, as it aggregates and updates the features of each node’s neighboring nodes to obtain a new representation of the node. When stacking multiple edge convolution layers, the model can capture deeper and farther distance hand surface point cloud features. Each layer of edge convolution further refines and integrates features based on the previous layer, enabling the model to gradually learn richer and more complex hand surface point cloud features. As the number of layers increases, the model can more accurately describe the entire gesture.

However, the number of edge convolution layers is not the more the better. When there are too many edge convolution layers, the performance of the model will actually decrease. This is because excessive edge convolution layers can make the features between neighboring information too smooth when aggregating the neighborhood information of the central point. In this process, originally discriminative features are excessively fused, resulting in a weakening of the uniqueness and differences of the features, making the features between different gestures similar and lacking sufficient discriminability. In this way, it is difficult for the model to accurately distinguish different gestures during gesture recognition, resulting in a decrease in recognition accuracy.

In addition, we can see that the accuracy changes obtained by different experimenters not only have consistent trends, but also the maximum accuracy values occur when the number of edge convolution layers is 3, achieving a relatively high level of performance. Furthermore, further increasing the number of edge convolution layers does not bring significant performance improvement. Increasing the number of edge convolution layers will increase the complexity of the model, as well as the number of parameters and computational complexity. Therefore, it is reasonable to choose an appropriate number of edge convolution layers.

### 3.3 Multi perspective analysis of dimensional abstraction enhancement processing results

**Figure 6:**
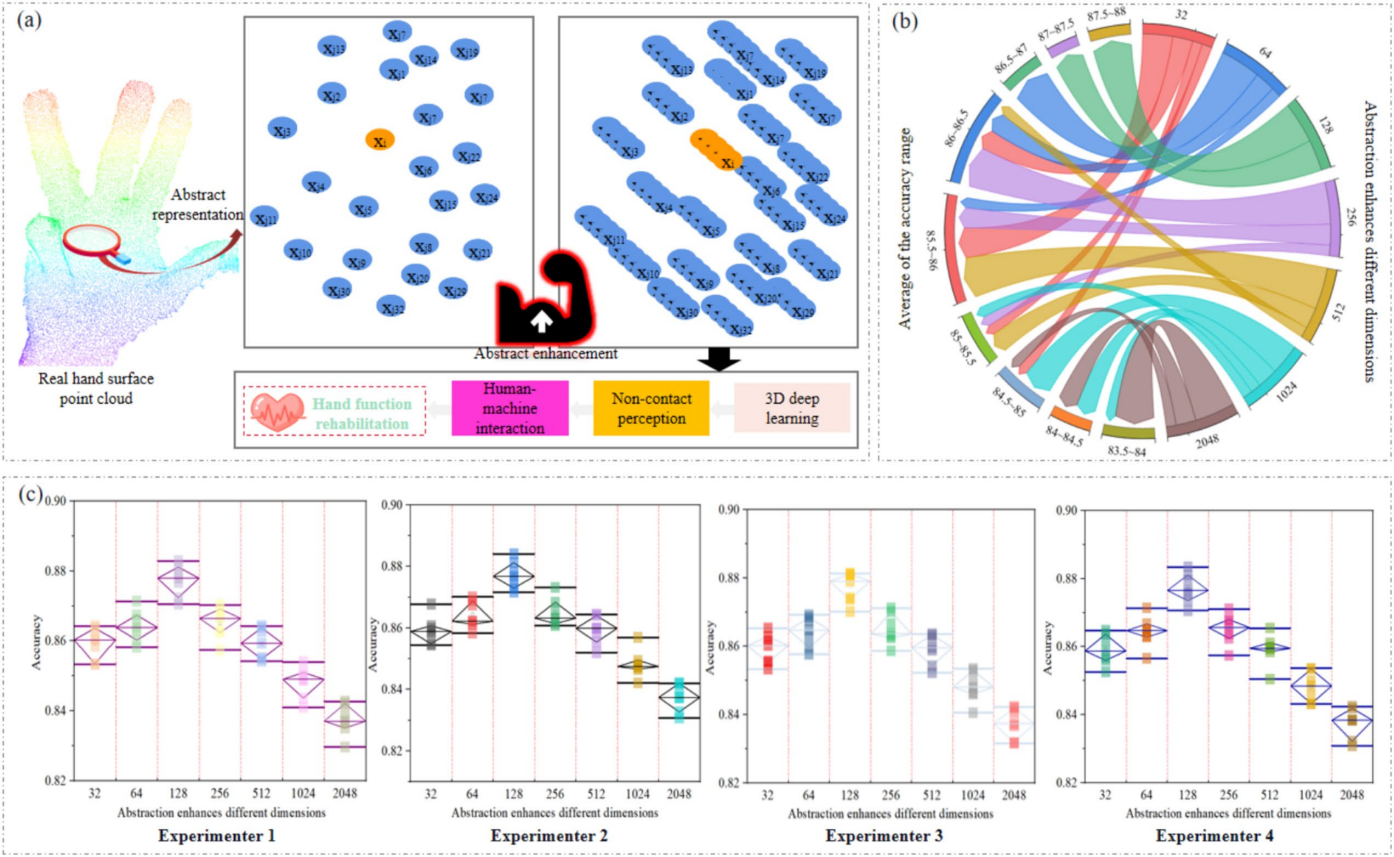
**(a)** Abstract enhancement of dimension of hand surface point cloud. **(b)** Chord diagrams of multiple repeated experiments conducted by all experimenters in different dimensions. **(c)** The experimental results of each experimenter in different dimensions.

Abstract enhancement of the dimension of the hand surface point cloud can enable the 3D deep learning model to learn the features of the point cloud more accurately, as shown in Figure 6a. We designed multiple experiments to increase the dimensions of the hand surface point cloud to 32, 64, 128, 256, 512, 1,024, and 2,048. In each experiment, we used the same training and testing sets, and trained and evaluated them under the same network parameter configuration. At the same time, we analyzed the results obtained by four experimenters under different dimensions of abstraction enhancement. In order to ensure the comprehensiveness and objectivity of the experiment, each experimenter conducted multiple repeated experiments, as shown in Figure 6b. The experimental results of each experimenter in different dimensions are shown in Figure 6c.

The abstract enhancement of different dimensions has a significant impact on the performance of hand surface point cloud recognition methods. As the dimensions increase, the performance of the model shows a trend of first improving and then decreasing. Specifically, as the dimension gradually increases from 32, the accuracy of hand surface point cloud recognition gradually improves. This is because when the dimensions are small, the model’s representation ability is limited, and it cannot fully capture and represent the advanced features of the hand surface point cloud. The hand surface point cloud contains rich information, and models in low dimensions find it difficult to effectively integrate and express this information, resulting in lower recognition accuracy. With the increase of dimensions, the representation ability of the model is enhanced, and it can handle more complex features, thereby improving the accuracy of gesture recognition. However, when the dimension reaches a certain threshold, the situation changes. Continuing to increase dimensions, the improvement in performance is no longer significant, and there may even be a decline. This is because when the dimensions are too large, the model becomes increasingly complex, overfitting the training data during the training process, ultimately leading to a decrease in accuracy.

Based on the experimental results, we found that when the dimension is around 128, the accuracy of hand surface point clouds is higher, and the model can fully capture the advanced features of hand surface point clouds. Besides, enhancing dimensions is like adding more attributes to data. The original hand surface point cloud only had simple information such as position, but after adding features, more abstract descriptions were added to the hand surface point cloud. This enables GSFAN to more accurately recognize different gestures and achieve non-contact gesture surface feature analysis in human-machine interaction.

### 3.4 Multi perspective analysis of different cutting-edge models

**Figure 7:**
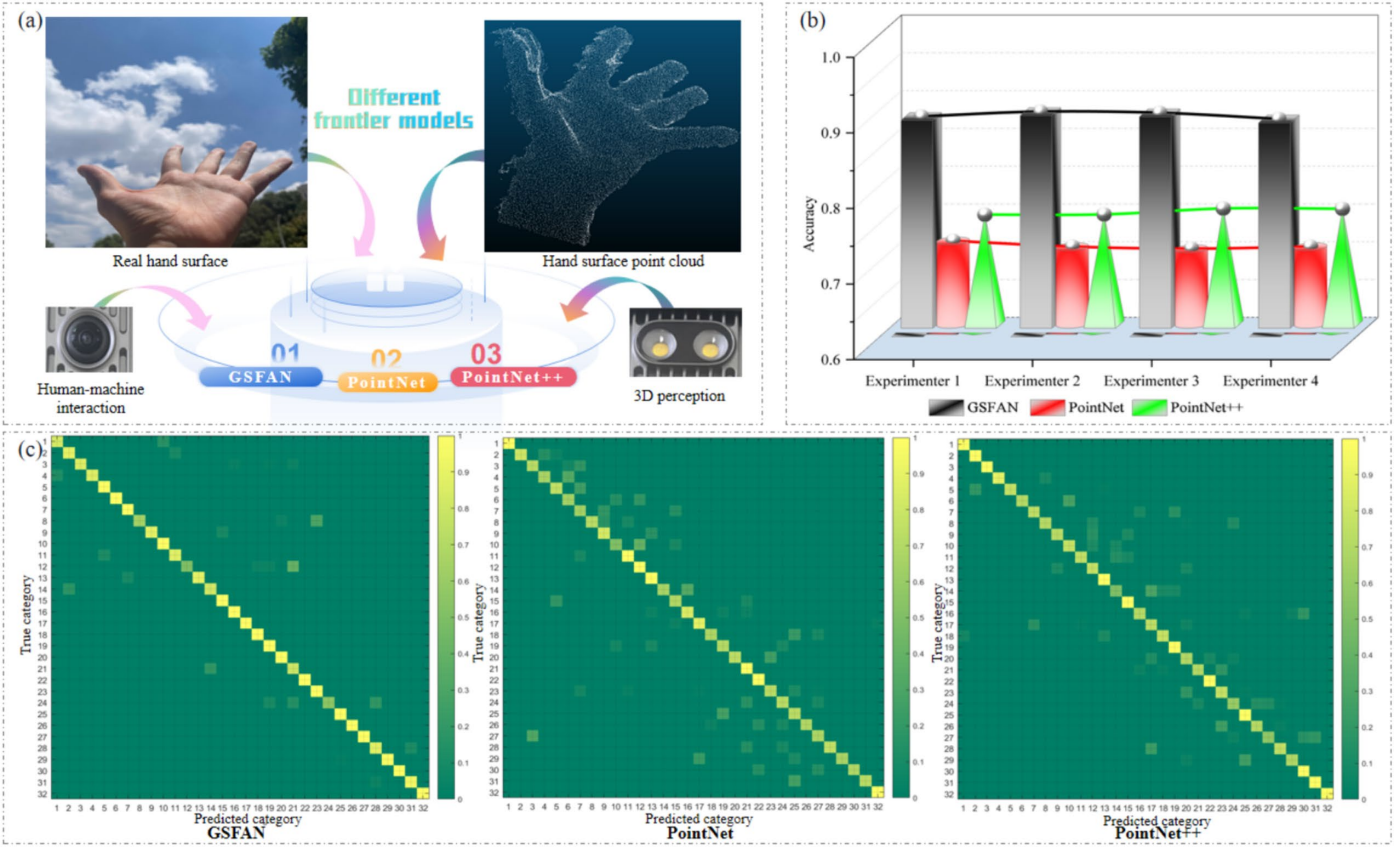
**(a)** Processing hand surface point clouds using different cutting-edge models. **(b)** Overall accuracy of different cutting-edge models on hand surface point clouds of different experimenters. **(c)** Confusion matrix of different cutting-edge models on second experimenter’s hand surface point clouds.

In order to verify the progressiveness and superiority of the model we built in this research, we will analyze the results obtained by GSFAN, PointNet, PointNet++ in processing point clouds on the hand surface of different experimenters (Figure 7a). For fair comparison, we trained and tested in the same hardware and software environment, using the same dataset and evaluation metrics. The overall accuracy of different cutting-edge models on the hand surface point clouds of different experimenters is shown in Figure 7b. The confusion matrix can observe the performance of the model on various categories. We analyze the confusion matrix of different cutting-edge models in the second experiment and evaluate the performance of different cutting-edge models on different gesture categories. Figure 7c shows the confusion matrix of 32 hand surface point clouds obtained from different cutting-edge models, where the horizontal axis represents the predicted results, the vertical axis represents the true results, and the diagonal matrix represents the recognition accuracy of each hand surface point cloud. The reason for choosing accuracy as the evaluation metric is that it can intuitively reflect the performance of the model in the overall gesture recognition task, and in actual hand function rehabilitation application scenarios, users are more concerned about whether the model can accurately recognize gestures, thereby ensuring the effectiveness of rehabilitation training.

In the comparative experiment, we observed that the GSFAN established in this research had an overall accuracy better than PointNet and PointNet++for different experimenters, indicating that this model has higher accuracy in hand surface point cloud processing tasks. In addition, different cutting-edge models have certain sensitivity to the hand surface point clouds provided by different experimenters, and the results obtained may fluctuate and change. This is mainly because the hand surface point clouds of each experimenter cannot be completely consistent, but the accuracy obtained by GSFAN can still reach a high level, with an average accuracy of 88.72%. And the above experimental results also demonstrate that GSFAN can still achieve good results and has good robustness when facing different hand surface point clouds given by experimenters.

In order to conduct a more in-depth analysis of the performance of each model, we further plotted the confusion matrix. The confusion matrix can clearly demonstrate the recognition ability of the model on various categories, especially on easily confused hand surface point cloud categories. It can be seen that for the 32 types of hand surface point clouds, GSFAN is able to distinguish each type of point cloud well and has good classification performance. PointNet and PointNet++have a high false recognition rate on certain specific hand surface point cloud categories. Through comparative analysis, we found that the GSFAN established in this research exhibits superior performance in hand surface point cloud processing tasks. This is mainly due to the following aspects: the GSFAN constructed in this research obtains local information of the hand surface point cloud through edge convolution, which can more effectively capture the spatial structure and local detail features of gestures. Moreover, GSFAN increases the dimensional information of the gesture point cloud, which can further extract advanced feature representations of the hand surface point cloud, thereby improving the model’s discriminative ability. In contrast, although PointNet and PointNet++can handle unordered point cloud data, they have certain limitations when dealing with hand surface point cloud data. PointNet is unable to capture local detail features in point clouds. As an improved version of PointNet, although PointNet++has improved the ability to extract local features to a certain extent, it still has not escaped the constraints of PointNet in essence, and still appears inadequate when dealing with complex hand surface point clouds.

Through the above experiments and result analysis, we can conclude that the model established in this research has good performance in hand surface point cloud processing tasks. This provides a new approach and method for non-contact analysis of gesture surface features in human-machine interaction in the field of hand function rehabilitation.

In traditional hand function rehabilitation scenarios, rehabilitation methods often rely on physical contact and manual guidance. Patients need to perform various rehabilitation actions with the assistance of therapists, and the rehabilitation process is usually mechanically repeated according to established procedures, which makes the entire rehabilitation process appear dull and boring. Moreover, manual guidance has certain limitations, such as the difficulty for therapists to accurately monitor the quality and subtle changes of each patient’s movements at all times. High accuracy gesture recognition enables patients to interact with rehabilitation equipment in a more natural and convenient way. Patients only need to make gestures, and rehabilitation equipment can accurately recognize and provide corresponding feedback without the need for tedious operations. The natural interaction greatly enhances the patient’s rehabilitation experience and makes the rehabilitation process more enjoyable. Positive psychological experiences can greatly stimulate patients’ rehabilitation enthusiasm, making them more willing to actively participate in rehabilitation training, thereby improving rehabilitation outcomes.

In addition, during the rehabilitation process, therapists need to accurately determine whether patients can complete specific rehabilitation training and adjust the training intensity and methods in a timely manner. High accuracy gesture recognition can provide reliable reference for therapists. Therapists can gain a clear understanding of patients’ hand movements based on relevant feedback, which helps them develop more scientific and personalized rehabilitation plans, and improve the efficiency and quality of rehabilitation treatment.

In order to better adapt the model to the characteristics of each individual, we plan to introduce personalized training mechanisms in the future. In practical applications, users perform a series of simple gestures, and we use this data to fine tune the pre trained model. By fine-tuning, the model can adjust parameters based on the individual characteristics of new users, thereby better recognizing their gestures. This personalized training mechanism can improve the recognition accuracy of the model for each individual without affecting the overall performance of the model. In addition to the features of the hand surface point cloud itself, we will also consider integrating other relevant features, such as the user’s basic information (age, gender, etc.) and the physiological characteristics of the hand (hand size, finger length, etc.). These additional features can provide the model with more information about individual differences, helping the model better understand and adapt to the characteristics of different individuals. Meanwhile, we will introduce an adaptive adjustment mechanism in the model to enable it to automatically adjust decision rules based on the characteristics of input data. For example, when the model detects that the input data is similar to an individual feature in the training data, it can automatically adjust the corresponding weights to improve recognition accuracy.

This research has limitations in some aspects. Firstly, we used laser sensors to collect hand surface point cloud, but this hardware has certain limitations. On the one hand, the accuracy of laser sensors is limited, which may result in inaccurate point cloud data collection and loss of some subtle features on the hand surface. On the other hand, the collection range of laser sensors is also limited. When the hand exceeds its effective range, some point cloud data will be missing, which will affect subsequent feature extraction and model training. Secondly, in this research, the number of samples used for model training and testing was relatively small. This is mainly due to the fact that the data collection process requires a significant amount of time and labor costs. A small sample size may lead to insufficient generalization ability of the model, making it difficult to adapt well to gesture changes in different individuals and environments. For example, there may be differences in hand size among different individuals, and small sample data may not cover all of these changes, resulting in poor performance of the model when faced with new data. Finally, the current research mainly focuses on the accuracy of gesture recognition, but lacks validation of the real-time performance of the model. In practical human-machine interaction and rehabilitation application scenarios, real-time performance is a very important indicator. Patients need to receive timely feedback in order to adjust their actions. However, due to the high computational complexity of the model, it can cause delays in recognition results and fail to meet real-time requirements. In the future, we will research lightweight deep learning architectures to reduce the computational complexity of models.

## 4 Conclusion

In the current context of the interweaving trend of normalized epidemic prevention and control and aging population, this research focuses on the field of intelligent rehabilitation of hand function in human-machine interaction. A 3D deep learning model was constructed to process laser sensor point clouds and achieve non-contact gesture surface feature analysis. Through this innovative method, we are not only able to accurately recognize and analyze the surface features of gestures, but also have achieved significant breakthroughs in the security and convenience of human-machine interaction. Firstly, this research effectively captured the spatial structure and local detail features of gestures through edge convolution. Through experiments, it was found that the model performed best when the number of edge convolution layers in GSFAN was 3. Secondly, abstraction enhances the dimensional information of the hand surface point cloud, which can further extract advanced feature representations of the hand surface point cloud. Through experiments, it was found that the recognition accuracy of the hand surface point cloud is higher when the dimension is 128. Finally, this research verified the progressiveness and superiority of GSFAN through comparative experiments. Compared with the cutting-edge point cloud processing model, GSFAN showed higher accuracy and lower confusion rate when classifying 32 hand surface point clouds. The average accuracy of GSFAN is 88.72%. The results of this research indicate that our proposed model has stronger application potential and practical value in the field of intelligent rehabilitation of hand function in human-machine interaction.

In addition, in the subsequent research work, we will conduct extensive research on cutting-edge research achievements in the field of point cloud processing, select representative and influential advanced models, and carry out systematic comparative experiments. During the experiment, we will strictly control the experimental conditions to ensure the scientificity and reliability of the comparative results. By comparing the key indicators of different models in hand surface point cloud segmentation tasks, comprehensively evaluate the performance and advantages of GSPAN. At the same time, we will conduct in-depth analysis and discussion of the experimental results, exploring the advantages and disadvantages of different models in processing hand surface point cloud.

## Data Availability

The original contributions presented in the study are included in the article/supplementary material, further inquiries can be directed to the corresponding authors.
